# Quality of pharmacy services and adherence to good pharmacy practice at points of sale in Punjab, Pakistan: A cross-sectional study

**DOI:** 10.1371/journal.pone.0348798

**Published:** 2026-05-20

**Authors:** Jamshaid Akbar, Atif Usman, Imran Masood, Ali Hassan Gillani, Muhammad Ahmad Mahmood, Zainub Khan, Muhammad Yousif, Ali Ahmed

**Affiliations:** 1 Department of Pharmacy Practice, Faculty of Pharmacy, The Islamia University of Bahawalpur, Bahawalpur, Pakistan; 2 Office of Research, Innovation and Commercialization, The Islamia University of Bahawalpur, Bahawalpur, Pakistan; 3 Department of Pharmacy Administration and Clinical Pharmacy, School of Pharmacy Xi’an Jiaotong University, Xi’an, Shaanxi, China; 4 Center for Drug Safety and Policy Research, Xian Jiaotong University, Xi’an, Shaanxi, China; 5 Shaanxi Center for Health Reform and Development Research, Xi’an, Shaanxi, China; 6 Health and Population Department, Government of Punjab, Lahore, Pakistan; 7 Akbar Institute of Medical Sciences, Bahawalpur, Pakistan; 8 Division of Infectious Diseases and Global Public Health, School of Medicine, University of California San Diego (UCSD), La Jolla, California, United States of America; 9 Perelman School of Medicine, University of Pennsylvania, Philadelphia, Pennsylvania, USA; Universiti Sains Malaysia, PAKISTAN

## Abstract

Adhering to good pharmacy practice (GPP) guidelines ensures the safe, effective, and ethical delivery of pharmaceutical services at community pharmacies, commonly referred to as Point of Sale (POS). However, developing countries such as Pakistan experience poor compliance with GPP. This study aimed to evaluate the quality of pharmacy services and adherence to GPP, focusing on technology compliance, infrastructure, legal aspects, dispensing practices, storage conditions, and other essential facilities. A descriptive cross-sectional study design was employed for the current study. The sample size was determined using the online sample size calculator Raosoft^TM^. The method adopted for the distribution of sample among selected cities was done by proportional sampling and the convenience sampling technique was employed for accessing individual POS. A self-designed questionnaire, consisting of five sections, i.e., i) demographic data, ii) prescription recording and data management system, iii) pharmacy infrastructure, facilities and services, iv) dispensing, preparation, administration and v) distribution of medicine and storage facilities was used to gather data from the selected POS of Punjab, Pakistan between 20^th^ July 2024–20^th^ September 2024. The data were entered and analysed using IBM SPSS 23.0. Descriptive and inferential statistics were used to describe the results. A total of 598 POS were included in the final analysis. Approximately 90.6% were independent POS, with 72.7% operating in the A category. A qualified person (QP) was observed in only 29.1% of POS. Approximately 31.9% of POS did not have any computerized technology, 52.2% lacked a system for monitoring the facility’s temperature, and a significant proportion (84.6%) were involved in prescribing for minor ailments. The mean score was higher in POS operating under category A licenses, chain setups and those with one or more QP. The highest significant (*p* < 0.05) mean difference observed between chain and independent POS was 3.53 (95% CI = 2.44, 4.64), between category A licensing type and no available license was 11.99 (95% CI = 8.13, 15.84), and between the presence and absence of a QP was 2.30 (95% CI = 1.58, 3.01). The findings revealed that the lack of adherence to GPP is a common practice in the country and is significantly linked to various factors such as POS type, type of license of POS, and availability and number of QP at the POS. To enhance compliance with GPP, the collaborative efforts of various stakeholders, along with targeted interventions, are essential.

## Introduction

With an ever-growing population and the incorporation of advancements in life-saving disease management, the burden on the healthcare system is overwhelming [1]. To facilitate overburdened well-recognized healthcare professionals, i.e., physicians and nurses, a significant change within the healthcare team has been the addition of allied health professionals, including pharmacists [2], physiotherapists [3], dietitians and nutritionists [4]. Within these professionals, pharmacists have the most diverse playable role; they can work in hospital, clinical, and community settings playing crucial role in managing medications, preventing disease, and educating patients [2,5,6]. Developed nations understood this importance swiftly, upgraded their curricula, reformed their rules, legislation and regulations, and incorporated pharmacists as an accountable member of the healthcare team [7–9]. In contrast, the development of the pharmacy profession has lagged in low- and middle-income countries (LMICs), where the pharmacy profession is still overlooked and under recognized that is especially true in the context of community pharmacist [10–14].

Traditionally, the role of the community pharmacist was considered to be only preparing and dispensing pharmaceutical products [15]. The idea was true when chemists were considered drug experts, and the establishment was referred to as a medical store. However, advancements in the healthcare system shifted pharmacists’ focus from product-oriented services to patient-oriented services [11,16]. The introduction of the Doctor of Pharmacy (Pharm-D) program is one such initiative strengthening this role, for example, in countries such as the United States (US) and Canada, diploma-level pharmacy qualifications have primarily been replaced by advanced professional degrees, i.e., Pharm-D and B-Pharm, respectively [9,17]. The recognised degree in these developed countries is a 4–5-year degree program, after which individuals can apply for a license to operate a pharmacy [17]. Licenses are issued only once pharmacists successfully pass the competency exam and adhere to the established guidelines [9]. This step replaced the term’ medical store’ with ‘pharmacy’ in developed countries, along with the concept of unqualified technicians or dispensers [8,18,19]. The scenario is relatively different in developing countries, where the concept of medical stores and unqualified dispensers is still prevalent [20]. Pharmacies and medical stores are still collectively referred to as a Point of Sale (POS) in LMICs. The variation in POS operations lies in the qualified professional’s (QP) license issued by the regulatory authority of the respective region. For instance, in Pakistan, those holding a Pharm-D degree are granted a pharmacy license and are called Category-A qualified professionals (QP-CA), while those with a diploma in pharmacy technician are given a medical store license and are known as Category-B qualified professionals (QP-CB). The operating systems and hierarchies of these POS, however, differ across countries, with particularly notable differences observed between developing and developed nations [21].

The World Health Organization (WHO) and the International Pharmaceutical Federation [22,23] collaborated to standardize and enhance the role of pharmacists. This effort resulted in the development of GPP guidelines, widely acknowledged as the standard defining the expected quality of services offered by pharmacies [22]. Comprehensively, the GPP framework precisely outlines each detail of a POS, including adopting the latest digital technologies, proper storage and dispensing of medication, patient management and counselling, as well as the pharmacy’s infrastructure and layout [24–27]. Several tools have been developed to assess compliance with GPP [28–30]. Implementing the findings of these tools yields significant results in enhanced adherence to GPP and an overall improvement in the quality of pharmacy services.

Several studies have been conducted to assess pharmacy services in Pakistan [31–38]. However, none of these gave a detailed exploration before; most of the studies mainly focused on the legal aspects of POS operations and a couple of them partially explored the dispensing practices, yet none of the studies focused on all the aspects of GPP. To fill the said gap, the current study aimed to evaluate the quality of pharmacy services and adherence to GPP of POS in Pakistan. It focuses on technology compliance, infrastructure, legal aspects, dispensing practices, storage conditions, and other essential facilities that will provide a valuable perspective on the POS-driven hindrance towards compliance with GPP guidelines. The study also intends to identify areas for improvement, both in infrastructure and professional practices, that hinder the integration of pharmacists into the broader healthcare system and enhance the quality of patient care.

## Materials and methods

### Study design and setting

A descriptive cross-sectional study design was utilized in this study following STROBE guidelines (**S1 File** STROBE Statement—Checklist of items that should be included in reports of *cross-sectional studies*) [39]. It included various POS in selected cities of Punjab, Pakistan. Punjab is the second largest and most densely populated province, known for its robust healthcare system, characterised by a complex network of pharmacies and medical stores [40,41].

### Sample size determination

The total number of valid POS was obtained from the Punjab Centralized Drug Sale Licensing (CDSL) public portal [42] accessed on July 02, 2024. Appropriate filters were used to exclude facilities located in areas beyond the study’s scope and those that are distribution-based POS. The resulting figure was 36,296, which was used to calculate the sample size using the online sample size calculator Raosoft^TM^ with a 99% confidence interval, a 5% margin of error, and a 50% response distribution. The obtained sample size was 654; considering a 10% attrition rate, the final number reached 719. This was employed to calculate the strata for each selected city based on the proportion of valid number of POS. The final sample obtained was Bahawalpur 70, DG Khan 33, Lahore 278, Faisalabad 141, Rawalpindi 125, and Sialkot 72.

### Data collection tool

The data collection tool was self-designed through an extensive literature review [25,37,43–48] and was evaluated for both content and face validity. To ensure the questions’ quality and relevance, five field experts were extensively consulted during the questionnaire’s development. This group included two academicians (FH & SA) specializing in social and administrative pharmacy research, two representatives (AA & IJ) from regulatory authorities, and one (RS) from the linguistic department. Reliability was assessed through pilot testing (not included in the final analysis), with a Cronbach’s alpha coefficient of 0.789, indicating acceptable consistency. The finalised questionnaire consisted of 5 distinct sections (A to E) with 110 items and a maximum score of 96 (S2 File Data collection tool). Section A, i.e., outcomes of interest, consisted of 14 items capturing the sociodemographic characteristics of the participants. Variables of interest were captured in Section B through E comprising of items evaluating GPP. Section B consisted of 15 items examining the POS prescription recording and management, and Section C assessed the POS infrastructure, facilities, and services with 26 items. Section D covered the dispensing, preparation, and administration of medicines through 31 items and Section E evaluated storage facilities within the POS and comprised of 24 items. Except for section A, all items were on a dichotomous scale (Yes or No), with every correct response marked 01 and incorrect ones marked 0.

### Data collection procedure

Data collection was conducted over a period of two months, from 20^th^ July 2024 to 20^th^ September 2024. Before initiating data collection, formal written approval was obtained from the Chief Drugs Controller’s Office (CDC) in Punjab, via letter number NO.CDC/GL-1/2024, followed by verbal approvals from the Drug Controller’s Office in each respective area. POS were selected based on accessibility, location, and willingness to participate, which was ensured by obtaining informed verbal consent from each participating individual. The principal investigator administered the questionnaire to the QP if they were present at that time. In instances where a QP was unavailable, the questionnaire was administered to the most senior salesman present during the visit. Efforts were made to visit the POS independently, without any representatives from the local drug control office, to reduce potential bias.

### Data analysis

Data analysis was performed using the Statistical Package for the Social Sciences (SPSS), Version 23. Descriptive statistics were applied to summarise the data in terms of frequencies (n), percentages (%), mean (∑) and standard deviation (±SD). Inferential statistics, including a one-way ANOVA test, were applied to determine the difference in mean scores between multiple independent groups. An independent sample T-test was used to compare the means of two independent groups on the outcomes of interest, testing for statistically significant differences. For all the statistical tests, the alpha was set at p < 0.05, the confidence interval CI at 95% and the margin of error at 5%.

### Ethical consideration

The research project was approved by the Pharmacy Research and Ethics Committee of Islamia University of Bahawalpur, vide Ref. No. 219-2024/PHEC, Dated April 15, 2024. Moreover, participation was voluntary, and all participants were informed about the study’s objectives, methods, and potential risks, and were allowed to withdraw at any time. Participant anonymity was guaranteed throughout the research.

## Results

**Fig 1:**
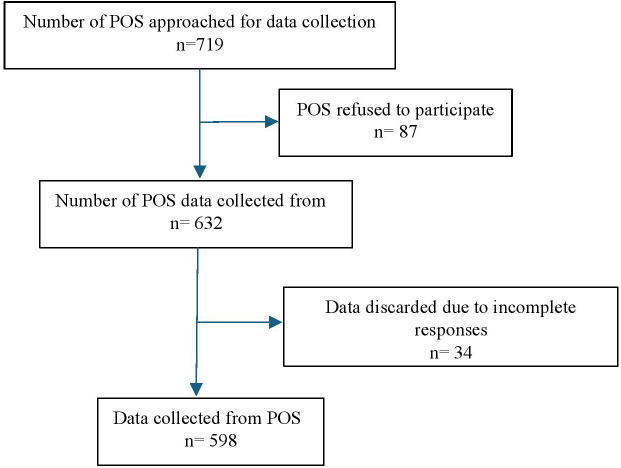
Process of POS selection and inclusion.

Out of 719 POS, 87 chose not to participate in the study because they did not consent to share their information, resulting in a response rate of 87.9% with 632 samples. Responses from 34 POS were further removed during data cleaning because they were incomplete, with less than 50% of the form filled out. As a result, the study was conducted on 598 POS (Fig 1).

The majority (90.6%) were independent POS with 72.7% operating in category A. With a mean number of sales staff of 4.74 (±3.34), the presence of a QP was observed in only 29.1% of the POS at the time of visit. Nearly half of the POS respondents reported that their QPs worked for 1–8 hours daily, and only 33.1% of setups reported having a full-time pharmacist (Table 1). The overall number of POS compliant with the developed GPP investigation tool, along with the distribution of mean scores based on the classification of outcomes of interest (S3 File Raw results). Raw data used for the analysis is also attached in S4 File.

**Table 1:** Demographic Data of Point of Sale (POS).

Outcomes of Interest		n (%) / ∑ (SD)
**Demographic Data**		
City Name	Rawalpindi	111 (18.6)
	Faisalabad	109 (18.2)
	Sialkot	68 (11.4)
	Lahore	212 (35.5)
	Bahawalpur	67 (11.2)
	D G Khan	31 (5.2)
Type of Point of Sale (POS)	Independent	542 (90.6)
	Chain	56 (9.4)
Total Operating Hours	< 5	8 (1.3)
	6–15	313 (52.3)
	> 15	277 (46.3)
Years in Business	10.62 (8.83)	
Type of License	Category A	435 (72.7)
	Category B	139 (23.2)
	Any other	17 (2.8)
	Not available	7 (1.2)
Presence of Qualified person (QP)	Yes	174 (29.1)
	No	424 (70.9)
Number of QP	No QP	168 (28.1)
	At least 1 QP	341 (57.0)
	More than 1 QP	89 (14.9)
Working hours of QP	0 hours	168 (28.1)
	1-8 hours	262 (43.8)
	>8 hours	168 (28.1)
Nature of job of QP	POS owner	114 (19.1)
	Business partner	22 (3.7)
	Full-time pharmacist	198 (33.1)
	Part-time pharmacist	264 (44.1)
Experience of QP (Years)	<5	327 (54.7)
	6–10	165 (27.6)
	>10	106 (17.7)
Total number of sales staff	4.75 (3.34)	
Qualification of sales staff (Senior most)	Under matric	135 (22.6)
	Matric – Intermediate	251 (42.0)
	Above Intermediate	212 (35.5)
Field of last qualification of sales staff	Medical Sciences	308 (51.5)
	Non-medical Sciences	290 (48.5)
What is the average number of patients dispensed per day at the pharmacy	<100	193 (32.3)
	100–300	252 (42.1)
	>300	153 (25.6)

### Mean scores

The mean score for each section, i.e., section B through E, was calculated against demographic variables of section A, i.e., outcomes of interest, to examine variations based on the POS characteristics and is presented in Table 2. The mean score of POS in section B was (5.74 ± 3.55) from a total score of 15. The highest mean was for chain POS (8.37 ± 1.64) and POS with more than 1 QP (7.88 ± 1.91), followed by those with a full-time pharmacist (7.14 ± 2.82). Section C had a total score of 26, with an average POS score of (11.40 ± 4.17). POS with more than 1 QP and a full-time pharmacist both scored highest in their categories (13.56 ± 2.36 and 13.56 ± 3.15). POS without a license scored lowest (7 ± 2.70). Section D’s total score was 31, with a mean score of (12.5 ± 3.18). Facilities without a license had the lowest score (9.57 ± 1.61), while those with a full-time pharmacist scored highest (14.01 ± 2.91). The mean score for section E was (15.83 ± 4.40) out of 24, with the highest score for POS with QP (17.01 ± 4.13). The presence of more than one QP significantly improved storage facilities with a mean score of (18.4 ± 2.72).

**Table 2:** Mean scores.

Outcomes of Interest		∑ (SD) of scores attained by grouped POS in respective section			
Section A: Demographic Data		B	C	D	E
City (Name)	Rawalpindi	4.98 (3.42)	10.69 (3.60)	12.00 (3.38)	16.44 (3.98)
	Faisalabad	5.06 (4.00)	10.44 (4.83)	11.96 (3.18)	14.88 (5.47)
	Sialkot	4.27 (3.74)	9.05 (4.78)	10.82 (2.73)	15.75 (3.98)
	Lahore	7.14 (2.38)	12.82 (2.39)	13.25 (2.52)	16.26 (4.20)
	Bahawalpur	5.82 (4.01)	9.16 (3.38)	11.46 (2.52)	13.85 (3.66)
	D G Khan	4.29 (4.33)	17.67 (3.74)	17.09 (3.38)	18.51 (2.79)
Type of Point of Sale (POS)	Independent	5.47 (3.58)	11.07 (4.17)	12.37 (3.21)	15.62 (4.47)
	Chain	8.37 (1.64)	14.60 (2.38)	13.83 (2.61)	17.85 (2.99)
Total Operating Hours	< 5	6.12 (3.83)	11.75 (3.57)	13.87 (2.69)	15.50 (3.20)
	6–15	4.70 (3.59)	10.61 (4.43)	12.15 (3.20)	14.53 (4.65)
	> 15	6.90 (3.12)	12.28 (3.69)	12.88 (3.14)	17.31 (3.60)
Type of License	Category A	6.68 (3.05)	12.02 (4.03)	12.82 (3.20)	16.84 (3.75)
	Category B	3.56 (3.69)	10.05 (4.27)	11.94 (3.04)	13.96 (4.47)
	Any other	1.76 (2.16)	8.41 (1.97)	10.41 (2.34)	9.70 (3.34)
	Not available	0.28 (0.48)	7.00 (2.70)	9.57 (1.61)	4.85 (3.71)
Presence of Qualified person (QP)	Yes	6.83 (3.03)	13.40 (3.62)	14.07 (2.94)	17.01 (4.13)
	No	5.29 (3.65)	10.73 (4.20)	11.87 (3.06)	15.35 (4.42)
Number of QP	No QP	3.94 (3.49)	9.75 (3.93)	11.03 (2.56)	14.33 (5.07)
	At least 1 QP	6.07 (3.50)	11.66 (4.34)	12.78 (3.26)	15.91 (4.08)
	More than 1 QP	7.88 (1.91)	13.56 (2.36)	14.23 (2.80)	18.34 (2.72)
Working hours of QP	O hours	3.94 (3.49)	9.75 (3.93)	11.03 (2.56)	14.33 (5.07)
	1-8 hours	6.02 (3.50)	11.48 (4.37)	12.35 (3.22)	16.23 (4.00)
	>8 hours	7.10 (2.91)	12.95 (3.42)	14.22 (2.88)	16.71 (3.89)
Nature of job of QP	POS owner	5.18 (3.83)	11.41 (4.22)	13.35 (3.20)	15.05 (4.75)
	Business partner	6.86 (3.80	13.31 (6.11)	13.95 (3.88)	17.81 (3.94)
	Full-time pharmacist	7.14 (2.82)	13.56 (3.15)	14.01 (2.91)	17.73 (3.10)
	Part-time pharmacist	4.83 (3.55)	9.62 (3.79)	10.89 (2.50)	14.57 (4.56)
Experience of QP (Years)	<5	6.33 (3.27	11.99 (3.77)	12.66 (3.02)	16.35 (4.03)
	6–10	6.15 (3.53)	11.54 (4.67)	12.76 (3.50)	16.10 (4.75)
	>10	3.26 (3.37	9.37 (3.92)	11.62 (3.03)	13.80 (4.38)
Qualification of sales staff	Under matric	3.48 (3.74)	10.51 (4.92)	12.45 (3.56)	13.91 (4.87)
	Matric – Intermediate	5.66 (3.51)	11.16 (4.57)	12.42 (3.44)	15.76 (4.49)
	Above Intermediate	7.27 (2.54)	12.26 (2.78)	12.65 (2.56)	17.14 (3.41)
Field of last qualification of sales staff	Medical Sciences	6.81 (2.88)	12.20 (3.47)	12.88 (2.90)	16.96 (3.57)
	Non-medical Sciences	4.60 (3.83)	10.55 (4.66)	12.11 (3.41)	14.63 (4.86)
What is the average number of patients dispensed per day at the pharmacy	<100	3.55 (3.67)	10.64 (4.83)	12.12 (3.47)	14.32 (4.90)
	100–300	6.34 (3.29)	11.71 (3.95)	12.59 (3.13)	15.85 (4.21)
	>300	7.49 (2.20)	11.86 (3.43)	12.84 (2.85)	17.69 (3.15)
**Mean / total score**		**5.74 (3.55) / 15**	**11.40 (4.17) / 26**	**12.51 (3.18) / 31**	**15.83 (4.40) / 24**

### Difference in mean scores

The mean score differences are shown in Table 3. In Section B, a significant difference was found in the mean scores of POS type, with p < 0.001, showing the highest difference between chain and independent POS (2.90 ± 0.48). For QP demographics, the greatest mean difference was between POS with more than one QP versus those without any QP (3.94 ± 0.43, p < 0.01). Section C indicates the highest difference between POS holding category A and those with no license (5.03 ± 1.54). In Section D, a higher score difference was seen among type A POS and those lacking a license (3.25 ± 1.19, p = 0.034). Additionally, a significant difference (3.20 ± 0.39, p < 0.001) was found between POS with more than one QP and those without QP. Section E shows a notable difference between category A POS and those without any license (11.99 ± 1.49, p < 0.001).

**Table 3:** Difference in mean scores.

Sections		B			C			D			E		
Outcomes of Interest		Difference (SE)	95% CI	P value	Difference (SE)	95% CI	P value	Difference (SE)	95% CI	P value	Difference (SE)	95% CI	P value
**Point of Sale** ^**†**^	Chain vs Independent	2.90 (0.48)	1.953, 3.865	< 0.001	3.53 (0.57)	2.414, 4.645	< 0.001	1.46 (0.44)	0.593, 2.336	0.001	2.23 (0.61)	1.031, 3.432	< 0.001
**Operating Hours** ^**†**^	Above 15 (hours) vs 6–15 (hours)	2.20 (0.28)	1.540, 2.852	< 0.001	1.67 (0.33)	2.463, 0.875	< 0.001	0.73 (0.26)	0.117, 1.345	0.015	2.77 (0.34)	1.962, 3.585	< 0.001
**License Type** ^**†**^	Cat A vs Cat B	3.12 (0.31)	2.323, 3.920	< 0.001	1.97 (0.39)	0.956, 2.984	< 0.001	0.87 (0.30)	0.083, 1.658	0.023	2.88 (0.38)	1.899, 3.869	< 0.001
	Cat A vs Any other	4.91 (0.78)	2.891, 6.944	< 0.001	3.62 (0.99)	1.043, 6.188	0.002	2.41 (0.77)	0.411, 4.407	0.011	7.14 (0.97)	4.643, 9.642	< 0.001
	Cat A vs Not available	6.39 (1.21)	3.274, 9.519	< 0.001	5.03 (1.54)	1.064, 8.992	0.006	3.25 (1.19)	0.169, 6.328	0.034	11.99 (1.49)	8.139, 15.843	< 0.001
	Cat B vs Not available	3.28 (1.23)	0.101, 6.450	0.040	**–**	**–**		**–**	**–**	**–**	9.11 (1.52)	5.190, 13.023	< 0.001
	Cat B vs Any other	**–**	**–**	**–**	**–**	**–**		**–**	**–**	**–**	4.26 (1.01)	1.660, 6.856	< 0.001
	Any other vs Not available	**–**	**–**	**–**	**–**	**–**		**–**	**–**	**–**	4.85 (1.76)	0.308, 9.389	0.031
**Presence of Qualified Person (QP)** ^**‡**^	QP present vs QP absent	1.55 (0.31)	0.930, 2.163	<0.001	2.30 (0.36)	1.587, 3.017	< 0.001	2.20 (0.27)	1.669, 2.739	<0.001	1.66 (0.39)	0.893, 2.427	< 0.001
**Number of QP** ^**†**^	More than 1 QP vs No QP	3.94 (0.43)	2.925, 4.968	<0.001	3.81 (0.52)	2.581, 5.042	< 0.001	3.20 (0.39)	2.271, 4.129	<0.001	4.01 (0.55)	2.713, 5.316	< 0.001
	More than 1 QP vs At least 1 QP	1.81 (0.39)	0.889, 2.744	<0.001	1.89 (0.47)	0.781, 3.016	< 0.001	1.44 (0.35)	0.603, 2.290	<0.001	2.43 (0.50)	1.248, 3.611	< 0.001
	At least 1 QP vs No QP	2.12 (0.31)	1.395, 2.864	<0.001	1.91 (0.37	1.027, −2.797	< 0.001	1.75 (0.28)	1.085, 2.421	<0.001	1.58 (0.39)	0.648, 2.520	< 0.001

†Denotes ANOVA was conducted to ascertain the group’s differences while an Independent T-test was used in case of ^‡^.

## Discussion

In accordance with the GPP guidelines, the current study assessed the quality of pharmacy services provided by the POS of Punjab, Pakistan, within the country’s clinical and legal framework. Several issues have been identified in following GPP, including improper use of technology and record keeping, poor dispensing processes, inadequate patient counselling and subpar storage of medicines. Nonetheless, the most pressing issue was the unavailability of QP, i.e., pharmacist at POS. Globally, the requirement for a QP to be present at the POS is a regulatory and public health challenge. In many developed countries, this is strictly enforced, and a POS cannot operate without the presence of QP, and additionally, QP provides different types of health care services at POS [37,49,50]. The findings of the current study support this, as a significant difference in mean scores was observed in the POS where QP was serving, and the presence of more than one QP significantly improved the results.

The absence of QP does not align with the guidelines of the GPP and the country’s national and provincial laws. The results are unsurprising and conform to related research [51,52]. The absence of QP in community settings can be attributed to several factors, predominantly low salaries [53]. Furthermore, QP are reluctant to offer their services for a low monthly income [54]. Instead, they prefer other areas of the pharmacy profession, such as academia and industry, to build their careers. The findings of this study support these implications, as the majority of the pharmacists were serving as part-time workers at the POS. Even though the extended operating hours of QP are linked with quality care and services, as depicted by the notable difference in the mean scores. Most of QP had rented out their categories (where the pharmacists and technicians lease out their licenses to business owners and investors for a predetermined financial compensation), which is the unfortunate scenario [14]. These findings are in accordance with other studies from different regions [12,55,56].

A key factor in evaluating pharmacy service quality is how well a POS handles data and maintains records. Even though the world has adopted the new technologies and incorporated digital things [57,58], it was observed in the current study that many POS still lack digitalization and are dependent on the conventional system of data keeping. This could be linked to the lack of appropriate knowledge about computerized technologies, the undertraining of staff, and even the unavailability of QP-CA in this regard [59–61]. Moreover, high prices, lower affordability, and power supply issues also make digitalization a strenuous task in Pakistan [62,63]. Another notable and interesting finding was that, even when present, digital record-keeping was mainly focused on managing stocks and the expiry of medicines, rather than patients’ healthcare record management. Although it was not identifiable whether QP-CA presence made an impact on such lack of interest, it remains another aspect the POS shall work out on.

In addition to maintaining patient and prescription records, keeping a record of certain medications is also essential and even mandatory, particularly for high-risk medicines [45]. A register must be maintained for proper record-keeping and signed by QP daily to facilitate this. Our study findings showed that many POS had a register to document the sale and purchase of narcotics and controlled medicines. However, only a small proportion had it routinely signed by QP, which is the primary duty outlined in Pakistan’s drug laws [14,45]. Neglecting this responsibility indicates noncompliance with regulations and highlights weaknesses in the regulatory framework of the pharmaceutical field. In the developed countries, pharmacies keep detailed logs, especially for controlled substances, supported by regular audits and digital systems [64]. Conversely, many LMICs countries [35,65,66] struggle with challenges like insufficient infrastructure, untrained personnel, and weak regulatory oversight, which lead to poor documentation practices [67]. Failure to have an updated record directly translates to compromised patient safety and higher chances of medication errors [68]. Such practices are also commonly observed in Pakistan and significantly undermine the quality of GPP, as depicted in the results of the current study. It also limits the basic function of a pharmacist to get engaged in patient care directly.

The marginal involvement and interaction between pharmacists and other healthcare providers affect dispensing protocols, as depicted by the current study’s results. A limited number of prescriptions were checked for possible interactions and adverse effects, and practically no one consulted the relevant physicians. No adequate referral system was observed in the majority of the POS. The general practitioners’ behavior and the healthcare system’s hierarchical landscape may be the root causes of this [69]. Almost all responding POS unanimously agreed on selling medications without the prescription of authorized medical practitioner, showcasing the poor compliance with the GPP guidelines. The current findings aligned with the similar study conducted in Karachi, Pakistan [34]. It reflects that the patients in general are in a two-way loss, i.e., neither the physician nor the pharmacist has an interest to help the patient keep them at a therapeutic goal, thereby increasing the risk of health deterioration, simultaneously reducing their health-related quality of life.

In addition to the roles defined for a QP, another important aspect in complying with GPP is maintaining a good storage of medicines at a POS. The current study illustrated that most POS were well equipped with satisfactory storage systems and hygienic conditions. These findings deviate from the study conducted in Karachi, Sindh, [34,70,71] since Punjab is relatively more developed than any other province of Pakistan. However, the power backup and 24/7 cooling system were absent in a significant number of POS in current study as well. Since load-shedding and power breakdown are common in Pakistan, it negatively impacts the POS environment despite the rules that require continuous uninterrupted power supply to assure delivery of efficacious medicine. Another factor is the sky-high rates of electricity bills and taxes [72] that prevent pharmacy owners from having ideal storage and temperature maintenance facilities. The storage facilities of category A POS were found to be much better as validated by the higher mean score, compared to category B POS and POS with no operating licence. This may be attributed to the fact that category A POS are legally permitted to stock, store, and dispense a broader range of medicines than other categories of POS. Nonetheless, the current study indicates that maintaining GPP cannot be achieved solely by a qualified professional, a systematically operated establishment/POS, or the regulators alone, unless all of these entities work together to ensure the safe and efficacious use of medicines by patients.

## Conclusion

The current findings concluded that POS across Punjab, Pakistan, adhered to GPP guidelines and followed specific regulatory standards to some extent. Based on our understanding of the results, the presence of QP is one of the integral components in successfully implementing GPP, as the findings showed that the adherence to GPP was significantly higher, especially in settings where more than one QP was available to supervise services. Moreover, POS with longer opening hours, specifically those operating as part of chain pharmacies, demonstrated better compliance with GPP. Notably, the higher average scores in such POS emphasize the vital role of QP supervision, organizational capacity, and resource allocation in delivering quality pharmacy services. The study clarifies the current situation of pharmacy services in Pakistan and establishes a strong foundation for further exploration into the reasons behind the systemic barriers leading to non-compliance with GPP.

## Limitations of the study

The current study has some limitations. First, convenience sampling was used, which may have introduced selection bias and affected the representativeness of the sample to the broader population. Secondly, the findings may not fully reflect practices across all POS in Punjab or other parts of Pakistan. Although the study included six cities in Punjab, the results should be interpreted with caution when applying them to rural areas or the entire country.

## Supporting information

S1 File STROBE Statement—Checklist of items that should be included in reports of cross-sectional studies. (DOC)

S2 File Data collection tool. (DOCX)

S3 File Raw results. (DOCX)

S4 File Raw data used for analysis. (CSV)
