# Supplementary material for: Quality of pharmacy services and adherence to good pharmacy practice at points of sale in Punjab, Pakistan: A cross-sectional study
Source: PLoS One. 2026 May 20;21(5):e0348798. doi: 10.1371/journal.pone.0348798 (PMC13189300; doi:10.1371/journal.pone.0348798)
Supplement: S1 File — (DOC) [file pone.0348798.s001.doc]

STROBE Statement—Checklist of items that should be included in reports of ***cross-sectional studies***

|  | Item No | Recommendation | Response |
| --- | --- | --- | --- |
| **Title and abstract** | 1 | (*a*) Indicate the study’s design with a commonly used term in the title or the abstract | Page number 1 |
|  |  | (*b*) Provide in the abstract an informative and balanced summary of what was done and what was found | Pages 2-3 |
| Introduction | | |  |
| Background/rationale | 2 | Explain the scientific background and rationale for the investigation being reported | Pages 4-5 |
| Objectives | 3 | State specific objectives, including any prespecified hypotheses | Page number 6 |
| Methods | | |  |
| Study design | 4 | Present key elements of study design early in the paper | Page number 6 |
| Setting | 5 | Describe the setting, locations, and relevant dates, including periods of recruitment, exposure, follow-up, and data collection | Pages 6 -7 |
| Participants | 6 | (*a*) Give the eligibility criteria, and the sources and methods of selection of participants | Pages 7-8 |
| Variables | 7 | Clearly define all outcomes, exposures, predictors, potential confounders, and effect modifiers. Give diagnostic criteria, if applicable | Page number 7 |
| Data sources/ measurement | 8* | For each variable of interest, give sources of data and details of methods of assessment (measurement). Describe comparability of assessment methods if there is more than one group | Pages 6-8 |
| Bias | 9 | Describe any efforts to address potential sources of bias | Page number 8 |
| Study size | 10 | Explain how the study size was arrived at | Page number 6 |
| Quantitative variables | 11 | Explain how quantitative variables were handled in the analyses. If applicable, describe which groupings were chosen and why | Page number 8 |
| Statistical methods | 12 | (*a*) Describe all statistical methods, including those used to control for confounding | Pages 8-9 |
|  |  | (*b*) Describe any methods used to examine subgroups and interactions | Pages 15-18 |
|  |  | (*c*) Explain how missing data were addressed | Page number 9 |
|  |  | (*d*) If applicable, describe analytical methods taking account of sampling strategy | NA |
|  |  | (*e*) Describe any sensitivity analyses | NA |
| Results | | |  |
| Participants | 13* | (a) Report numbers of individuals at each stage of study—eg numbers potentially eligible, examined for eligibility, confirmed eligible, included in the study, completing follow-up, and analysed | Page number 9 |
|  |  | (b) Give reasons for non-participation at each stage | Page number 9 |
|  |  | (c) Consider use of a flow diagram | Figure 1 on page number 9 |
| Descriptive data | 14* | (a) Give characteristics of study participants (eg demographic, clinical, social) and information on exposures and potential confounders | Pages 9 & 10 |
|  |  | (b) Indicate number of participants with missing data for each variable of interest | NA |
| Outcome data | 15* | Report numbers of outcome events or summary measures | Pages 11-18 |
| Main results | 16 | (*a*) Give unadjusted estimates and, if applicable, confounder-adjusted estimates and their precision (eg, 95% confidence interval). Make clear which confounders were adjusted for and why they were included | Pages 10-18 |
|  |  | (*b*) Report category boundaries when continuous variables were categorized | Pages 11-13 |
|  |  | (*c*) If relevant, consider translating estimates of relative risk into absolute risk for a meaningful time period | NA |
| Other analyses | 17 | Report other analyses done—eg analyses of subgroups and interactions, and sensitivity analyses | All analysis explained |
| Discussion | | |  |
| Key results | 18 | Summarise key results with reference to study objectives | Pages 19 - 21 |
| Limitations | 19 | Discuss limitations of the study, taking into account sources of potential bias or imprecision. Discuss both direction and magnitude of any potential bias | Page number 23 |
| Interpretation | 20 | Give a cautious overall interpretation of results considering objectives, limitations, multiplicity of analyses, results from similar studies, and other relevant evidence | Pages 19 - 21 |
| Generalisability | 21 | Discuss the generalisability (external validity) of the study results | Page number 22 |
| Other information | | |  |
| Funding | 22 | Give the source of funding and the role of the funders for the present study and, if applicable, for the original study on which the present article is based | Page number 24 |

*Give information separately for exposed and unexposed groups.

**Note:** An Explanation and Elaboration article discusses each checklist item and gives methodological background and published examples of transparent reporting. The STROBE checklist is best used in conjunction with this article (freely available on the Web sites of PLoS Medicine at http://www.plosmedicine.org/, Annals of Internal Medicine at http://www.annals.org/, and Epidemiology at http://www.epidem.com/). Information on the STROBE Initiative is available at www.strobe-statement.org.
