# Supplementary material for: Quality of pharmacy services and adherence to good pharmacy practice at points of sale in Punjab, Pakistan: A cross-sectional study
Source: PLoS One. 2026 May 20;21(5):e0348798. doi: 10.1371/journal.pone.0348798 (PMC13189300; doi:10.1371/journal.pone.0348798)
Supplement: S2 File — (DOCX) [file pone.0348798.s002.docx]

**Quality of pharmacy services and adherence to good pharmacy practice at points of sale in Punjab, Pakistan: a cross-sectional study**

Facility code: **______________**

**DATA COLLECTION TOOL**

**ASSESSMENT OF QUALITY OF PHARMACY SERVICES IN PERSPECTIVE OF GOOD PHARMACY PRACTICE (GPP)**

**Section A: Demographic Data**

| A 1 | City (Name) | ________________ |
| --- | --- | --- |
| A 2 | Type of Point of Sale (POS) | 1. Independent 🞐 2. Chain 🞐 |
| A 3 | Total Operating Hours | ___________________ |
| A 4 | Years in Business | ___________________ |
| A 5 | Type of License | 1. Category A 🞐 2. Category B 🞐 3. Any other ____________ 4. Not Available 🞐 |
| A 6 | Presence of Qualified person (QP) (Observation at time) | 1. Yes 🞐 2. No 🞐 |
| A 7 | Number of QP (If Applicable) |  |
| A 8 | Working hours of QP |  |
| A 9 | Nature of job of QP | 1. POS owner 🞐 2. Business Partner 🞐 3. Full-time Pharmacist 🞐 4. Part-time Pharmacist 🞐 |
| A 10 | Experience of QP | _______________ Years |
| A 11 | Total number of sales staff |  |
| A 12 | Qualification of sales staff (Senior most) | 1. Under matric 2. Matric – Intermediate 3. Above Intermediate |
| A 13 | Field of last qualification of sales staff | 1. Medical Sciences 2. Non-Medical Sciences |
| A 14 | What is the average number of patients dispensed per day at the pharmacy?   1. ______________________ 2. Don’t Know | |

**Section B: Prescription Recording and Data Management System**

| B 1 | Does the POS have computer technology or computerized system installed? | Yes  🞐 | No  🞐 |
| --- | --- | --- | --- |
| B 2 | If Yes, Computerized system used for? (You can pick more than one answer)   1. Stock Management 🞐 2. FEFO (first expire first out) 🞐 3. Patient medication profiles 🞐 4. Labelling 🞐 5. Patient personal information 🞐 6. Recording prescriptions of controlled drugs 🞐 | | |
| B 3 | The Computerized System records (structure of record): (You can pick more than one answer)   1. Date 🞐 2. Patient name 🞐 3. Patient ID/CNIC 🞐 4. Name of prescriber 🞐 5. Name(s) of drug(s) prescribed 🞐 | | |
| **Statement** | | **Yes** | **No** |
| B 4 | Does the point of sale have register to record sale/purchase of controlled substance? | 🞐 | 🞐 |
| B 5 | If yes, is the register signed by QP on daily basis? | 🞐 | 🞐 |
| B 6 | Does QP keep copy of prescription of controlled substance? | 🞐 | 🞐 |

**Section C: Pharmacy Infrastructure, Facilities and Services**

| **Statement** | | **Yes** | **No** |
| --- | --- | --- | --- |
| C 1 | The front of the Point of sale is clearly inscripted “Pharmacy” or “Medical store”? | 🞐 | 🞐 |
| C 2 | License of the POS is clearly displayed on visible side? | 🞐 | 🞐 |
| C 3 | The name of the QP is clearly displayed? | 🞐 | 🞐 |
| C 4 | Glass door is present at the entrance of POS? | 🞐 | 🞐 |
| C 5 | The interior of the POS is dust free? | 🞐 | 🞐 |
| C 6 | The dispensing department is separated by a barrier to prevent the entry of public? | 🞐 | 🞐 |
| C 7 | Is there any arrangement for accessibility of disabled persons? | 🞐 | 🞐 |
| C 8 | Is there proper/appropriate seating available for patients? | 🞐 | 🞐 |
| C 9 | Availability of suitable place in POS to discuss confidential matters with patients and customers? | 🞐 | 🞐 |
| C 10 | Availability of drinking water for patients or customers? | 🞐 | 🞐 |
| C 11 | Availability of patient weight scale? | 🞐 | 🞐 |
| C 12 | Availability of patient height scale? | 🞐 | 🞐 |
| C 13 | Availability of patient education material/information sources? | 🞐 | 🞐 |
| C 14 | If yes, what type of education material/information sources available? (You Can pick more than one answer)   1. Patient Leaflets 🞐 2. News Letters 🞐 3. Medicine Handbooks 🞐 | | |
| C 15 | Availability of health screening services? | 🞐 | 🞐 |
| C 16 | If yes, which services are available?   1. Blood Pressure Checking 🞐 2. Blood Glucose Test 🞐 3. Prescription Glasses 🞐 4. Cholesterol Level 🞐 5. Vaccination 🞐 | | |
| C 17 | Involvement in health promotion activities? | 🞐 | 🞐 |
| C 18 | Involvement in generic substitution /switching? | 🞐 | 🞐 |
| C 19 | Prescribing for minor ailments? | 🞐 | 🞐 |
| C 20 | Availability of home delivery service? | 🞐 | 🞐 |

**Section D: Dispensing, Preparation, Administration and Distribution of Medicine**

| **Statement** | | **Yes** | **No** |
| --- | --- | --- | --- |
| D 1 | Have you ever checked the prescription for any mistake? | 🞐 | 🞐 |
| D 2 | Have you ever had to call back to the concerned physician? | 🞐 | 🞐 |
| D 3 | Do you dispense medicine without prescription? | 🞐 | 🞐 |
| D 4 | If yes, which drugs do you dispense without prescription?   1. NSAIDs 🞐 2. Antibiotics 🞐 3. Steroids 🞐 4. Hypertension related medications 🞐 5. Cardio Vascular disease related medication 🞐 6. Anti-diabetics and other endocrine related medications 🞐 7. Benzodiazepines and other anxiolytics 🞐 8. Gastrointestinal and endocrine medications🞐 9. Anti-depressants & anti psychotics 🞐 10. Neurological disease medication 🞐 11. Asthma and COPD medication 🞐 12. Smoking Cessation preparation 🞐 13. Vitamins & supplements 🞐 14. Cosmetic Preparations 🞐 15. Topical medication 🞐 16. Hormones/oral contraceptives 🞐 | | |
| **Statement** | | **Yes** | **No** |
| D5 | Do you provide the patient with enough information that aim at supporting adherence to treatment? | 🞐 | 🞐 |
| D 6 | Do you provide enough information to the patient that aim at reducing antimicrobial resistance by providing information about the appropriate use of antimicrobial drugs? | 🞐 | 🞐 |
| D 7 | Do you prepare any extemporaneous medicine preparations or medical products at the pharmacy? | 🞐 | 🞐 |
| D 8 | If yes, is the area specifically designated for the preparation of extemporaneous medicine preparations or medical products being used for that purpose and that purpose only? | 🞐 | 🞐 |
| D 9 | Do patients consult you for unusual responses to a medicine or a treatment? | 🞐 | 🞐 |
| D 10 | When a patient consults you for unusual responses to a medicine or a treatment, do you usually intervene or ask the patient to refer to their medical doctor/treating physician? | 🞐 | 🞐 |
| D 11 | What is the literature/bibliography resources available at the POS?   1. EDL 🞐 2. BNF 🞐 3. Medical Handbooks 🞐 4. Pharmacopeia 🞐 5. Internet access 🞐 | | |
| D 12 | Does the QP check every medicine prepared by staff before dispensing? | 🞐 | 🞐 |

**Section E: Storage Facilities**

| **Statement** | | **Yes** | **No** |
| --- | --- | --- | --- |
| E 1 | Is power supply provided to the pharmacy 24 hours a day? | 🞐 | 🞐 |
| E 2 | Is the pharmacy equipped with its own electric generator or Solar system? | 🞐 | 🞐 |
| E 3 | If yes, is the electric supply functional during the night? | 🞐 | 🞐 |
| E 4 | Is there any stock management system available that helps in maintaining level of stock/generating order? | 🞐 | 🞐 |
| E 5 | The stock management system helps in controlling product expiry date? | 🞐 | 🞐 |
| E 6 | Is there any system for the monitoring and periodic inspection of expiration dates of products and removal of outdated products? | 🞐 | 🞐 |
| E 7 | Is there any system available to return expired or unwanted medicines and medical devices to the POS? | 🞐 | 🞐 |
| E 8 | Are there any records for expired drugs? | 🞐 | 🞐 |
| E 9 | Are expired drugs stored separately? | 🞐 | 🞐 |
| E 10 | Is there any mechanism to control pest? | 🞐 | 🞐 |
| E 11 | Are medicines protected from direct exposure to sunlight? | 🞐 | 🞐 |
| E 12 | Is the room temperature/ambient temperature in the pharmacy monitored using a thermometer or any equivalent device? | 🞐 | 🞐 |
| E 13 | Is there a functional cooling system available in the POS? | 🞐 | 🞐 |
| E 14 | What is the cooling system used in the POS?   1. Fans 🞐 2. Air Conditioner 🞐 3. Air Cooler 🞐 4. Fans & Air Cooler 🞐 5. Fans & Air Conditioner 🞐 | | |
| E 15 | Is there a refrigerator available in the POS? | 🞐 | 🞐 |
| E 16 | Are only medicines stored in the refrigerator? | 🞐 | 🞐 |
| E 17 | Are items, other than medicines, stored in the refrigerator?  If yes, please specify __________________________ | 🞐 | 🞐 |
| E 18 | Is the temperature of the fridge monitored or recorded? | 🞐 | 🞐 |
| E 19 | All the monitoring devices and scales are calibrated? | 🞐 | 🞐 |
| E 20 | Expired, damaged or broken items are stored separately and properly labelled? | 🞐 | 🞐 |
| E 21 | Are there any leaks from pharmacy roof? | 🞐 | 🞐 |
| E 22 | Is the surface of the storage area sufficient to store all items on shelves (nothing on the floor)? | 🞐 | 🞐 |
| E 23 | Are the shelves properly labeled? | 🞐 | 🞐 |
| E 24 | Are controlled substances stored inside locked cupboards and/or locked drawers? | 🞐 | 🞐 |
