# Supplementary material for: Quality of pharmacy services and adherence to good pharmacy practice at points of sale in Punjab, Pakistan: A cross-sectional study
Source: PLoS One. 2026 May 20;21(5):e0348798. doi: 10.1371/journal.pone.0348798 (PMC13189300; doi:10.1371/journal.pone.0348798)
Supplement: S3 File — (DOCX) [file pone.0348798.s003.docx]

**Quality of pharmacy services and adherence to good pharmacy practice at points of sale in Punjab, Pakistan: a cross-sectional study**

**Supplementary file**

Table 1 displays the details of the POS prescription recording and data management system. About 31.9% of POS did not have computerized technology and the ones using computerized system, primarily used it for stock management. A significant number n=523 of POS maintained registers for recording the sale and purchase of controlled substances, but only 22.6% were signed daily by a qualified person.

Table 1: Prescription Recording and Data Management System

| **Variables of interest** | | **Response n (%)** | |
| --- | --- | --- | --- |
|  |  | **Yes** | **No** |
| Does the POS have computer technology or computerized system installed. | | 407 (68.1) | 191 (31.9) |
| If Yes, Computerized system used for | 1. Stock Management | 405 (67.7) | 193 (32.3) |
|  | 1. FEFO (first expire first out) | 360 (60.2) | 238 (39.8) |
|  | 1. Patient medication profiles | 30 (5.0) | 568 (95.0) |
|  | 1. Labelling | 8 (1.3) | 590 (98.7) |
|  | 1. Patient personal information | 63 (10.5) | 535 (89.5) |
|  | 1. Recording prescriptions of controlled drugs | 207 (34.6) | 391 (65.4) |
| The Computerized System records (structure of record) | 1. Date | 403 (67.4) | 195 (32.6) |
|  | 1. Patient name | 347 (58.0) | 251 (42.0) |
|  | 1. Patient ID/CNIC | 86 (14.4) | 512 (85.6) |
|  | 1. Name of prescriber | 66 (11.0) | 532 (89.0) |
|  | 1. Name(s) of drug(s) prescribed | 361 (60.4) | 237 (39.6) |
| Does the point of sale have register to record sale/purchase of controlled substance. | | 523 (87.5) | 75 (12.5) |
| If yes, is the register signed by QP on daily basis. | | 135 (22.6) | 463 (77.4) |
| Does QP keep copy of prescription of controlled substance. | | 33 (5.5) | 565 (94.5) |

Table 2 provides an overview of the infrastructure, facilities and services of 598 POS involved in the current research. The front of approximately 9 out of 10 POS (89.8%), were inscribed with “pharmacy” or “medical store” and in 80.9%, license was clearly and visibly displayed. Most setups (69.4%) lacked a suitable space for confidential discussions and consultation with the pharmacist and only 29.8% offered home delivery services.

Table 2: Pharmacy Infrastructure, Facilities and Services

| **Variables of interest** | | **Response n (%)** | |
| --- | --- | --- | --- |
|  |  | **Yes** | **No** |
| The front of the Point of sale is clearly inscripted “Pharmacy” or “Medical store” | | 537 (89.8) | 61 (10.2) |
| License of the POS is clearly displayed on visible side | | 484 (80.9) | 114 (19.1) |
| The name of the QP is clearly displayed | | 339 (56.7) | 259 (43.3) |
| Glass door is present at the entrance of POS | | 536 (89.6) | 62 (10.4) |
| The interior of the POS is dust free | | 482 (80.6) | 116 (19.4) |
| The dispensing department is separated by a barrier to prevent the entry of public | | 461 (77.1) | 137 (22.9) |
| Is there any arrangement for accessibility of disabled persons | | 125 (20.9) | 473 (79.1) |
| Is there proper/appropriate seating available for patients | | 336 (56.2) | 262 (43.8) |
| Availability of suitable place in POS to discuss confidential matters with patients and customers | | 183 (30.6) | 415 (69.4) |
| Availability of drinking water for patients or customers | | 311 (52.0) | 287 (48.0) |
| Availability of patient weight scale | | 360 (60.2) | 238 (39.8) |
| Availability of patient height scale | | 110 (18.4) | 488 (81.6) |
| Availability of patient education material/information sources | | 59 (9.9) | 539 (90.1) |
| If yes, what type of education material/information sources available. | 1. Patient Leaflets | 39 (6.5) | 559 (93.5) |
|  | 1. Newsletters | 31 (5.2) | 567 (94.8) |
|  | 1. Medicine Handbooks | 13 (2.2) | 585 (97.8) |
| Availability of health screening services | | 426 (71.2) | 172 (28.8) |
| If yes, which services are available. | 1. Blood Pressure Checking | 398 (66.6) | 200 (33.4) |
|  | 1. Blood Glucose Test | 347 (58.0) | 251 (42.0) |
|  | 1. Prescription Glasses | 36 (6.0) | 562 (94.0) |
|  | 1. Cholesterol Level | 19 (3.2) | 579 (96.8) |
|  | 1. Vaccination | 10 (1.7) | 588 (98.3) |
| Involvement in health promotion activities | | 111 (18.6) | 487 (81.4) |
| Involvement in generic substitution /switching | | 514 (86.0) | 84 (14.0) |
| Prescribing for minor ailments | | 506 (84.6) | 92 (15.4) |
| Availability of home delivery service | | 178 (29.8) | 420 (70.2) |

Table 3 presents a concise overview of the dispensing, preparation, administration and distribution of medicine across various POS. More than half (56%) of the POS admitted of not checking prescription for any possible prescription errors or mistakes, and 63.5% acknowledged that they neither intervene nor refer patients to the physicians. A high proportion (91%) confirmed dispensing medicines without a prescription, amongst these NSAIDs were most commonly dispensed (90.3%), closely followed by antibiotics (86.6%).

Table 3: Dispensing, Preparation, Administration and Distribution of Medicine

| **Variables of interest** | | | **Response n (%)** | |
| --- | --- | --- | --- | --- |
|  |  |  | **Yes** | **No** |
| Have you ever checked the prescription for any mistake | | | 263 (44.0) | 335 (56.0) |
| Have you ever had to call back to the concerned physician | | | 91 (15.2) | 507 (84.8) |
| Do you dispense medicine without prescription | | | 544 (91) | 54 (9) |
| If yes, which drugs do you dispense without prescription | 1. NSAIDs | | 540 (90.3) | 58 (9.7) |
|  | 1. Antibiotics | | 518 (86.6) | 80 (13.4) |
|  | 1. Steroids | | 258 (43.1) | 340 (56.9) |
|  | 1. Hypertension related medications | | 504 (84.3) | 94 (15.7) |
|  | 1. Cardiovascular disease related medication | | 338 (56.5) | 260 (43.5) |
|  | 1. Anti-diabetics and other endocrine related medications | | 490 (81.9) | 108 (18.1) |
|  | 1. Benzodiazepines and other anxiolytics | | 261 (43.6) | 337 (56.4) |
|  | 1. Gastrointestinal and endocrine medications | | 485 (81.1) | 113 (18.9) |
|  | 1. Anti-depressants & anti psychotics | | 302 (50.5) | 296 (49.5) |
|  | 1. Neurological disease medication | | 276 (46.2) | 322 (53.8) |
|  | 1. Asthma and COPD medication | | 454 (75.9) | 144 (24.1) |
|  | 1. Smoking Cessation preparation | | 252 (42.1) | 346 (57.9) |
|  | 1. Vitamins & supplements | | 501 (83.8) | 97 (16.2) |
|  | 1. Cosmetic Preparations | | 456 (76.3) | 142 (23.7) |
|  | 1. Topical medication | | 511 (85.5) | 87 (14.5) |
|  | 1. Hormones/oral contraceptives | | 233 (39.0) | 365 (61.0) |
| Do you provide the patient with enough information that aim at supporting adherence to treatment | | | 439 (73.4) | 159 (26.6) |
| Do you provide enough information to the patient that aim at reducing antimicrobial resistance by providing information about the appropriate use of antimicrobial drugs | | | 198 (33.1) | 400 (66.9) |
| Do you prepare any extemporaneous medicine preparations or medical products at the pharmacy | | | 50 (8.4) | 548 (91.6) |
| If yes, is the area specifically designated for the preparation of extemporaneous medicine preparations or medical products being used for that purpose and that purpose only | | | 43 (7.2) | 555 (92.8) |
| Do patients consult you for unusual responses to a medicine or a treatment | | | 269 (45.0) | 329 (55.0) |
| When a patient consults you for unusual responses to a medicine or a treatment, do you usually intervene or ask the patient to refer to their medical doctor/treating physician | | | 218 (36.5) | 380 (63.5) |
| What is the literature/bibliography resources available at the POS | | 1. EDL | 11 (1.8) | 587 (98.2) |
|  |  | 1. BNF | 32 (5.4) | 566 (94.6) |
|  |  | 1. Medical Handbooks | 30 (5.0) | 568 (95.0) |
|  |  | 1. Pharmacopeia | 24 (4.0) | 574 (96.0) |
|  |  | 1. Internet access | 306 (51.2) | 292 (48.8) |
| Does the QP check every medicine prepared by staff before dispensing | | | 185 (30.9) | 413 (69.1) |

A significant percentage of POS (71.2%) were equipped with electric generators or solar systems and (76.8%) confirmed that the electric supply remained functional during the night. Refrigerators were present in almost all the setups (95.2%) and in 83.4% of the POS, medicines were protected from direct sunlight, but 52.2% lacked a system for monitoring temperature of the facility. Expired, damaged or broken items were stored and labelled separately in 76.4% pharmacies, yet only 39.5% kept controlled substances in locked cupboards or drawers (Table 4).

Table 4: Storage Facilities

| **Variables of interest** | **Response n (%)** | |
| --- | --- | --- |
|  | **Yes** | **No** |
| Is power supply provided to the pharmacy 24 hours a day | 447 (74.7) | 151 (25.3) |
| Is the pharmacy equipped with its own electric generator or Solar system | 426 (71.2) | 172 (28.8) |
| If yes, is the electric supply functional during the night | 459 (76.8) | 139 (23.2) |
| Is there any stock management system available that helps in maintaining level of stock/generating order | 465 (77.8) | 133 (22.2) |
| The stock management system helps in controlling product expiry date | 449 (75.1) | 149 (24.9) |
| Is there any system for the monitoring and periodic inspection of expiration dates of products and removal of outdated products | 282 (47.2) | 316 (52.8) |
| Is there any system available to return expired or unwanted medicines and medical devices to the POS | 339 (56.7) | 259 (43.3) |
| Are there any records for expired drugs | 225 (37.6) | 373 (62.4) |
| Are expired drugs stored separately | 508 (84.9) | 90 (15.1) |
| Is there any mechanism to control pest | 342 (57.2) | 256 (42.8) |
| Are medicines protected from direct exposure to sunlight | 499 (83.4) | 99 (16.6) |
| Is the room temperature/ambient temperature in the pharmacy monitored using a thermometer or any equivalent device | 286 (47.8) | 312 (52.2) |
| Is there a functional cooling system available in the POS | 564 (94.3) | 34 (5.7) |
| Is the cooling system as per requirement | 122 (20.4) | 476 (79.6) |
| Is there a refrigerator available in the POS | 569 (95.2) | 29 (4.8) |
| Are only medicines stored in the refrigerator | 465 (77.8) | 133 (22.2) |
| Are items, other than medicines, stored in the refrigerator | 171 (28.6) | 427 (71.4) |
| Is the temperature of the fridge monitored or recorded | 290 (48.5) | 308 (51.5) |
| All the monitoring devices and scales are calibrated | 158 (26.4) | 440 (73.6) |
| Expired, damaged or broken items are stored separately and properly labelled | 457 (76.4) | 141 (23.6) |
| Are there any leaks from pharmacy roof | 87 (14.5) | 511 (85.5) |
| Is the surface of the storage area sufficient to store all items on shelves (nothing on the floor) | 503 (84.1) | 95 (15.9) |
| Are the shelves properly labeled | 440 (73.6) | 158 (26.4) |
